# Campbell's Monkeys Use Affixation to Alter Call Meaning

**DOI:** 10.1371/journal.pone.0007808

**Published:** 2009-11-12

**Authors:** Karim Ouattara, Alban Lemasson, Klaus Zuberbühler

**Affiliations:** 1 Laboratoire EthoS “Ethologie animale et humaine”, U.M.R. 6552-C.N.R.S., Université de Rennes 1, Station Biologique, Paimpont, France; 2 Centre Suisse de Recherches Scientifiques, Abidjan, Côte d'Ivoire; 3 Laboratoire de Zoologie et de Biologie Animale, Université de Cocody, Abidjan, Côte d'Ivoire; 4 School of Psychology, University of St Andrews, St Andrews, Scotland, United Kingdom; University of California San Diego, United States of America

## Abstract

Human language has evolved on a biological substrate with phylogenetic roots deep in the primate lineage. Here, we describe a functional analogy to a common morphological process in human speech, affixation, in the alarm calls of free-ranging adult Campbell's monkeys (*Cercopithecus campbelli campbelli*). We found that male alarm calls are composed of an acoustically variable stem, which can be followed by an acoustically invariable suffix. Using long-term observations and predator simulation experiments, we show that suffixation in this species functions to broaden the calls' meaning by transforming a highly specific eagle alarm to a general arboreal disturbance call or by transforming a highly specific leopard alarm call to a general alert call. We concluded that, when referring to specific external events, non-human primates can generate meaningful acoustic variation during call production that is functionally equivalent to suffixation in human language.

## Introduction

Questions about the origins of human language and its potential precursors in animal communication remain controversial [1], [2]. A number of interesting parallels have been identified, such as babbling, audience effects, conversation-like interactions, or dialects, but the distribution of these phenomena is phylogenetically heterogeneous and often found in species that are not closely related to humans [3], [4]. Human language is highly complex and numerous characteristics appear to have no equivalent counterparts in animal communication systems. One such qualitative difference concerns the morpho-syntactic organisation of language, that is, the fact that morphological and syntactic elements are governed by a set of language-specific rules, the source of much of the generative power of human language [5], [6].

A number of recent field studies have demonstrated meaningful call combinations in the natural communication of non-human primates, such as putty-nosed monkeys [7], [8] or black and-white Colobus monkeys [9], [10]. Similarly, some species of birds, gibbons and whales have been observed to combine song elements into more complex utterances, in some cases recursively [11]–[14]. Birdsong in particular tends to have hierarchical and non-random transitional structure, and experimental change to its composition, rhythm, or component order tends to interfere with its communicative function [15]–[19]. Despite these examples of combinatorial signalling, there are no good examples in animal communication studies of individuals acoustically modifying individual calls in patterned ways to produce structurally altered vocalisations with novel meanings. In human speech, however, this process is ubiquitous. Human languages rely on numerous morphological processes to alter meaning, one prominent example being affixation, the addition of a morpheme (the smallest linguistic unit that has semantic meaning), to a word stem (the part of the word that never changes), as for instance in the English word ‘brother-hood’ [6]. Although non-human primates are able to discriminate between subtle acoustic changes in human speech signals [20], it is unknown whether they also produce such acoustic patterns as part of their natural communication.

Some of our previous studies with Campbell's monkeys have revealed an unusually high degree of vocal flexibility in various call types, often linked with social variables [21]–[25]. Females form the core of a social group and interact frequently with each other both physically and vocally [26]. Like other forest guenons, the single adult male remains spatially and socially peripheral but plays a key role in predator defence and coordination of travel [27]–[28]. Females produce a range of different call types, including distress, threat, contact, and warning calls [21–25; 29]. In contrast, males vocalise much less often and produce only a few loud call types, which function in spacing and predator defence [30]. In pilot observations, we have noted subtle but seemingly consistent acoustic variation is some of these calls, which suggested an affixation-like acoustic organisation. To address this point, we monitored the adult males of six wild Campbell's monkey groups in the Tai Forest, three of which were fully habituated to human observers. Data were collected both during the males' responses to naturally occurring disturbances and by simulating the presence of predators with visual and acoustic models.

## Results

### Call structure

**Figure 1:**
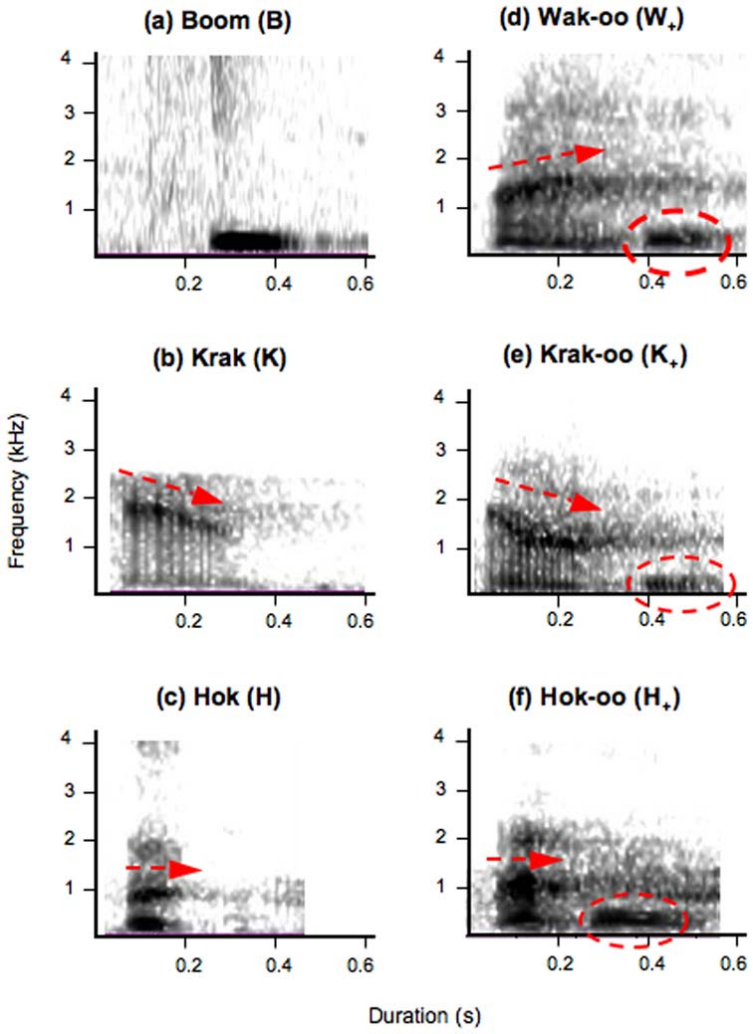
Spectrographic illustrations of the different loud call types produced by male Campbell's monkeys in different contexts. (a) ‘boom call’, a low-pitched loud call produced by the vocal sac with no frequency modulation, (b) ‘krak’ call [K], a single loud tonal utterance of ø = 0.176s duration, with a decreasing main frequency band starting at about 2.2 kHz; (c) ‘hok’ call [H], a single loud tonal utterance of ø = 0.070s with no frequency modulation starting at about 1.0 kHz); (d) ‘wak-oo’ call [W+], a suffixed loud tonal utterances of 0.330s consisting of a call stem with an increasing main frequency band rising from about 1.0 to 1.3 kHz, followed by a compulsory ‘oo’ suffix (e) ‘krak-oo’ call [K+], a ‘krak’ call followed by the ‘oo’ suffix; (f) ‘hok-oo’ [H+], a ‘hok’ call followed by the ‘oo’ suffix.

We found that, in all study groups, the adult males consistently produced six different loud alarm call types, “hok (Audio S1)”, “hok-oo” (Audio S2), “krak” (Audio S3), “krak-oo” (Audio S4), “wak-oo” (Audio S5), and “boom” (Audio S6), all of which were perceptually distinct to a human observer (fig. 1, see methods). “Boom” calls were much lower pitched than the other five loud calls, acoustically inflexible, always given in pairs with inter-call intervals of about seven seconds, and typically preceding a series of other loud calls [31]. The remaining five loud calls were acoustically more flexible. They differed from each other in the frequency contour of the call stem and, crucially, in whether or not the stem was trailed by an acoustically invariable “oo” utterance. In terms of frequency contours, the “krak” and “krak-oo” calls were characterised by a largely decreasing main frequency band, “hok” and “hok-oo” calls were mainly flat, while “wak-oo” calls had an increasing band (table 1; fig. 1).

**Table 1:** Basic acoustic measurements of the stem of the six different loud calls produced by adult male Campbell's monkeys.

Call	Mean duration (s) ± SE			Mean main frequency ± SE		
	Stem	“oo” suffix	Inter-unit	Beginning stem (Hz)	Transition stem (∂Hz)	“oo” suffix
**Boom**	0.095			159	0	
(N = 90)	±0.002**a**	–	–	±0.76**a**	±0.00**a**	–
**Wak-oo**	0.175	0.093	0.063	1061	-294	311
(N = 90)	±0.003**b**	±0.001**a**	±0.001**a**	±9.77**b**	±9.07**b**	±2.155**a**
**Krak**	0.185			2219	505	
(N = 224)	±0.001**b**	–	–	±19.16**c**	±8.45**c**	–
**Krak-oo**	0.182	0.098	0.064	1860	507	311
(N = 300)	±0.001**b**	±0.003**a**	±0.002**a**	±11.81**d**	±6.29**c**	±1.51**a**
**Hok**	0.079			988	00	–
(N = 171)	±0.004**c**	–	–	±10.75**e**	±0.00**a**	
**Hok-oo**	0.080	0.111	0.067	1020	00	307
(N = 168)	±0.001**c**	±0.009**a**	±0.004**a**	±10.83**e**	±0.00**a**	±1.78**a**

Duration of call stem: duration of the first section of krak-oo, wak-oo or hok-oo calls (excluding the affix) or the entire call for boom, krak or hok calls that carry no affixation. Transitions were calculated by subtracting the frequency at the beginning from the frequency at the end of the call or call stem. Results of Tukey post hoc tests for dyadic call comparisons: same letter  =  no significant difference; different letters  =  significant difference (p<0.001).

**Figure 2:**
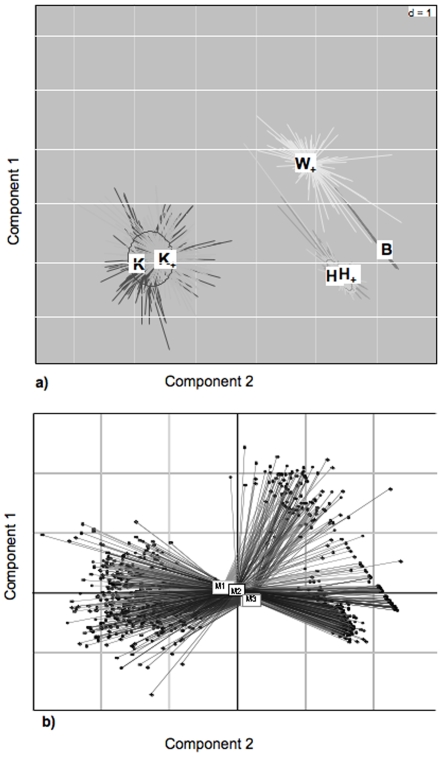
Results of the principal component analysis. (a) call stem clustering (b) male clustering. Call names (B, K, K+, W+, H and H+) are indicated at the corresponding position. M1, M2, M3  =  males 1–3.

We conducted a Pearson's based Principal Component Analysis to spatially display the different calls (fig. 2a). The total inertia was 94.54% with 78.94% for axis 1 (mainly driven by the start frequency of the call), and 15.60% for axis 2 (mainly driven by the duration of call stem), regardless of the identity of the caller (fig. 2b). Crucially, this analysis did not reveal any differences between the “krak” call and the stem of the “krak-oo” call, nor between the “hok” call and the stem of the “hok-oo” call. In addition, these four calls could be discriminated from the “boom” call and also from the stem of the “wak-oo” call (fig. 2a). We then conducted an analysis of variance, which revealed that the six calls differed significantly in the duration of the call stem (F_5, 1049_ = 549.58; p<0.001), the start frequency of the call stem (F_5, 1049_ = 9,199.46; p<0.001), and the transition frequency across the entire call stem (F_5, 1049_ = 512.17; p<0.001). Tukey post hoc tests failed to detect any significant differences in the key comparisons, that is, between the stems of “hok-oo” and “hok” and “krak-oo” and “krak” calls (table 1).

If a call contained a suffix (i.e. the “oo” unit), it was produced on average 0.060s following the stem, regardless of call type (ANOVA, df = 2, F_2,573_ = 0.207 p = 0.812). The “wak” stem was never produced singly, but always followed by the “oo” suffix to form the “wak-oo” call. For “krak” and “hok” calls, however, the “oo” suffix was optional. We then compared the acoustic structure of the “oo” units given in conjunction with the different stem calls and found no differences in terms of frequency or duration, indicating that callers produced only one acoustically invariant structure in this suffixed position (table 1).

### Call context

The three habituated males, who were tolerant to direct observations, produced their loud calls to a variety of disturbances, including both predatory and non-predatory events (table 2). GLM analyses, carried out with R-software, revealed that the production of the different call types varied according to context (main effects: context: LR Chi^2^ = 151.91, df = 8, p<0.0001; caller identity: LR Chi^2^ = 17.96, df = 2, p<0.0001; call type: LR Chi^2^ = 201.08, df = 5, p<0.0001; interaction effects: context x caller identity: LR Chi^2^ = 8.09, df = 13, p = 0.84; context x call type: LR Chi^2^ = 685.26, df = 40, p<0.0001; caller identity x call type: LR Chi^2^ = 28.40, df = 10, p<0.01; context x caller identity x call type: LR Chi^2^ = 27.65, df = 65, p = 0.99).

**Table 2:** Context-specificity of the different calls produced by the three habituated males in response to natural events.

Event	Male	N	Call type (mean N calls ± SD)					
			B	K	K_+_	H_+_	H	W_+_
Eagle attack	1	3	–	–	9±2	2±1	28±5	7±1
	2	2	–	–	6±1	2±1	41±8	3±1
	3	5	–	–	12±2	3±1	29±10	12±5
Sudden flying animal^1^	1	2	–	–	13±3	1±1	1±1	2±2
	2	2	–	–	16±3	–	–	1±1
Monkey eagle alarm calls^2^	1	3	–	–	13±5	6±2	2±2	3±2
	2	5	–	–	6±3	1±1	3±2	4±4
	3	6	–	–	11±8	2±2	1±1	5±3
Leopard encounter	1	1	–	13	8	–	–	–
	3	2	–	23±17	–	–	–	–
Terrestrial animal^3^	1	4	–	–	7±2	–	–	–
	2	4	–	–	3±1	–	–	–
	3	1	–	–	7	–	–	–
Fall of tree or branch	1	29	2±0	–	5±2	–	–	–
	2	5	2±0	–	8±2	–	–	–
	3	13	2±0	–	4±1	–	–	–
Neighbouring male^4^	1	12	2±0	–	6±3	3±1	–	–
	2	15	2±0	–	7±2	2±1	–	–
	3	27	2±0	–	8±2	3±2	–	–
Intense contact calling^5^	1	2	2±0	–	7±0	2±1	–	–
	2	10	2±0	–	8±2	3±1	–	–
	3	7	2±0	–	6±2	3±1	–	–
Group gathering and travelling^6^	1	4	2±0	–	–	–	–	–
	2	1	2	–	–	–	–	–
	3	5	2±0	–	–	–	–	–

B = boom, K_+_ = krak-oo; K = krak, H_+_ = hok-oo, H = hok, W_+_ = wak-oo. N = number of events; In each cell: mean (± standard deviation) number of calls recorded per event; ^1^mostly flying squirrels; ^2^mostly Diana monkeys, C. diana; ^3^mostly fleeing duikers (Cephalophus spp.); ^4^mostly male loud calls; ^5^Call exchanges by females and juveniles, usually in response to neighbouring male; ^6^Male usually out of visual contact with group.

Some calls were given to a broad, others to a narrow range of events. Crucially, “krak” calls were exclusively given after detecting a leopard, suggesting that it functioned as a leopard alarm call, whereas the “krak-oo” was given to almost any disturbance, suggesting it functioned as a general alert call. Similarly, “hok” calls were almost exclusively associated with the presence of a crowned eagle (either a real eagle attack or in response to another monkey's eagle alarm calls), while “hok-oo” calls were given to a range of disturbances within the canopy, including the presence of an eagle or a neighbouring group (whose presence could sometimes be inferred by the vocal behaviour of the females). On a few occasions, “hok” and “hok-oo” calls were produced in response to a flying squirrel, whose silhouette somewhat resembles a flying eagle, but never to any other large bird.

While producing “hok-oo” calls, males adopted a threat posture, combined with flashing their eyelids, and they sometimes conducted a short dash towards the disturbance. Although direct behavioural observations were only possible in 33.7% of calling events (N = 3 males; N = 83 events), we suspect that this kind of threat behaviour was common in conjunction with this call. None of the other calls was associated with such distinct behaviour, apart from “boom” calls that involved inflating the vocal sacs. Adding an “oo” unit to “hok”, thus, indicated that the male was aggressively motivated, and this was usually in response to a general disturbance that took place within the canopy, particularly a perched eagle or a conspecific opponent. “Wak-oo” calls were given to the same events as “hok-oo” calls (eagles, other flying animals, Diana monkey eagle alarms), but for some reason never to neighbours. “Boom” calls, finally, were only given to non-predatory contexts, such as a falling branch or tree, to initiate or halt group travel [see ref. 7], during disputes with neighbours, and during any unusual vocal excitation within the group (table 2).

To investigate the predator warning function more directly, we performed a series of field experiments that simulated the presence of the different predators, using both visual and acoustic models. Results confirmed our natural observations. Detecting a predator never triggered any “boom” calls. In contrast, the general alert call “krak-oo” was given in all four conditions, whereas “krak” calls were only produced in the presence of leopards. “Wak-oo” calls were given to eagles, while “hok” and “hok-oo” calls were primarily given to visual eagle models (table 3). General linear model analyses of variance revealed that the call rates of the different call types were significantly affected by the predator type, and by whether the caller could see the predator (table 4).

**Table 3:** Experimentally induced production of loud calls in 3 habituated and 4 semi-habituated males (B = boom, K_+_ = krak-oo; K = krak, H_+_ = hok-oo, H = hok, W_+_ = wak-oo).

Predator	Male	Call					
		B	K_+_	K	H_+_	H	W_+_
Eagle visual	1	–	10	–	5	11	13
	2	–	16	–	5	30	30
	3	–	25	–	4	26	22
	4	–	12	–	2	31	8
	5	–	–	–	6	15	6
	6	–	9	–	12	25	14
	7	–	19	–	3	13	7
Eagle acoustic	1	–	4	–	–	–	1
	2	–	1	–	1	4	4
	3	–	15	–	–	–	12
	4	–	12	–	–	–	4
	5	–	17	–	3	–	5
	6	–	1	–	3	5	2
	7	–	12	–	–	–	–
Leopard visual	1	–	–	48	–	–	–
	2	–	4	38	–	–	–
	3	–	–	29	–	–	–
	4	–	–	123	–	–	–
	5	–	–	15	–	–	–
	6	–	–	8	–	–	–
	7	–	–	12	–	–	–
Leopard acoustic	1	–	21	1	–	–	–
	2	–	6	8	–	–	–
	3	–	20	20	–	–	–
	4	–	5	–	–	–	–
	5	–	10	–	–	–	–
	6	–	5	–	–	–	–
	7	–	–	13	–	–	–

Each male was only exposed once to each model type.

**Table 4:** Results of GLM analysis of variance.

Call type		Caller	Predator	Modality
	Df	6, 18	1, 18	1, 18
Krak-oo (K_+_)	LR Chi2	32.94	98.52	5.53
	p	<0.001	<0.001	<0.05
Krak (K)	LR Chi2	156.61	388.16	−1.42_e_–14
	p	<0.001	<0.001	1
Hok-oo (H_+_)	LR Chi2	18.28	65.15	33.20
	p	<0.01	<0.001	<0.001
Wak-oo (W_+_)	LR Chi2	37.86	138.62	42.96
	p	<0.001	<0.001	<0.001
Hok (H)	LR Chi2	25.51	209.33	152.52
	p	<0.001	<0.001	<0.001

Generalized Linear Model analysis: Poisson distribution of error, log link function, type III.

## Discussion

We carried out long-term observations and predator model experiments to investigate how free-ranging male Campbell's monkeys of Taï National Park, Ivory Coast, communicated about external events. In previous research, we found that males and females produced different alarm calls that, in some cases, were combined into meaningful sequences [23], [31]-[34]. Here, we were interested in how acoustically flexible males were with some of their alarm call types, and how they applied this variation to external events. Our study showed that male Campbell's monkeys produced six different loud alarm calls in response to disturbing or dangerous events. “Boom” calls were acoustically and contextually unique, whereas the other five calls shared a number of acoustic features. The most relevant finding was that these five calls consisted of a call stem that differed in terms of the basic frequency contours and could be followed by an optional suffix-like small and inconspicuous vocal unit, which altered the semantic content of the full call in significant and predictable ways.

One important contrast between human and non-human primate vocal behaviour concerns the degree of motor control individuals have during call production. Humans are able to control their larynx and vocal tracts rapidly and precisely by means of various articulators, including tongue, mandible, and lips [35], [36]. The same basic mechanisms also play a role during vocal production in non-human primates, as illustrated by studies with Diana monkeys and other non-human primates [37]–[40]. Our results thus add to the growing literature that non-human primates use processes similar to the ones that are fundamental during speech production to communicate about events in their environment in a meaningful way.

The key finding of this study was that males adhered to a simple affixation rule, which increased their small basic vocal repertoire. “Krak” and “krak-oo” as well as “hok” and “hok-oo” calls were composed of the same call stem elements, while the rapid addition of the “oo” affix generated a significant change in the semantic content in terms of the types of external events the calls referred to. While “krak” and “hok” were predator-specific calls, the suffixed versions were produced in less specific contexts [31], [32]. We also conducted some pilot experiments during which we played back “hok-oo” and “krak-oo” calls to different groups of Diana monkeys, which often associate with Campbell's monkeys. As predicted, none of the tested Diana monkey groups showed any kind of significant anti-predator responses in these playbacks, in stark contrast to when hearing the non-suffixed Campbell's monkey alarm calls [31; unpublished data). We concluded that the Campbell's monkey alarm call system goes significantly beyond what has been described so far in the animal communication literature where acoustic diversity is normally achieved by modifications of frequency patterns, call rates, intensity differences, or sequential organisation [6], but not by suffixation.

The degree to which callers possess active control over their acoustic products is difficult to assess and it is also not clear what social factors influence call production in Campbell's monkeys and other primates. In blue monkeys, field experiments have shown that callers appear to take into account the degree to which other group members are at risk during eagle presence [41] and there is a growing literature of other types of audience effects that govern primate vocal behaviour. However, despite these results, it is still largely unclear whether non-human primates intentionally inform their audience about the event they have just experienced, or whether their vocal response is more directly driven by the psychological processes triggered by external events, the currently prevailing hypothesis. What our results show is that callers appear to make some judgements about the nature of the event (tree fall, group gathering to travel, conspecific intruder, eagle, leopard), and that this assessment determines whether or not affixation takes place. Equally important, male Campbell's monkeys rarely produce single calls but almost always give sequences of different call types [31]. Further research will have to address the role that affixation plays in these calling sequences in terms of context-specificity and whether listeners are able to comprehend the relationships between event and corresponding call sequence.

## Materials and Methods

The study was conducted in the Taï National Park (5°50′N, 7°21W), Ivory Cost, the largest remaining block of intact rainforest in West Africa. Data were collected between January 2006 and September 2007 on two groups of Campbell's monkeys (*Cercopithecus c. campbelli*) that were fully habituated to the presence of human observers. Campbell's monkeys routinely form polyspecific groups with other primates, particularly Diana monkeys, which whom they spend 77–89% of their time during feeding, travelling and resting [29]. Campbell's monkeys live in small one-male groups with 3–7 adult females with their offspring [23]. The two study groups have been followed on a regular basis since the early 1990s and all individuals can be recognised individually. We had additional access to four other groups that were partly habituated to human observers. During the study period, we observed one replacement of the single adult male in one habituated group. The new male became quickly habituated to human observers, which effectively increased the sample size of habituated individuals to N = 3.

Behavioural observations consisted of 15-min focal animal sampling and all-occurrence sampling. Three habituated males served as focal animal samples for a total of 40 hours (Male 1: 14 hours over 11 months, Male 2: 6 hours over 7 months; Male 3: 20 hours over 16 months). All occurrence sampling generated a total of about 2,000 observation hours, which lead to a total sample of 1,067 calls of acceptable acoustic quality for subsequent quantitative analyses. Under both data collection regimes, the observer (KO) recorded all vocalisations, the associated behaviour (travel, forage, rest, groom, aggression), and any unusual event immediately preceding a vocalisation, such as the presence of a leopard or crowned eagle, the calls of a neighbouring male, the thundering sound of a falling tree or large branch, the alarm calls of a nearby Diana monkey male to an eagle or terrestrial disturbance [33], the sudden appearance of an aerial or terrestrial non-predatory animal, or an unusually high rate of contact calls by female and juvenile group members. We also scored all events that directly followed a male call, particularly assemblies of dispersed group members or group travel.

Encounters with real predators were rare (N = 3 for leopards, *Panthera pardus*; and N = 11 for crowned eagle, *Stephanoaetus coronatus*) and we therefore conducted a series of field experiments during which we presented predator models, either by positioning a visual replica of the two main predators, or by broadcasting their typical vocalisations through a loudspeaker [8]. All seven different males were tested (3 habituated ones, 4 from the semi-habituated groups). Each male was exposed to a particular stimulus only once and the order of presentation of the different stimuli was randomised for each male. Before an experiment was carried out the following conditions had to be met: (a) the observer had to be in contact with the group for at least 30 min during which no alarm calls were produced; (b) the predator model (or playback speaker) had to be positioned by a field assistant on the projected travelling route. For eagle trials, the model or loudspeaker was positioned 2–3m off the ground. For leopard trials, it was positioned on the ground. Eagle shrieks were recorded in the study area by KZ; leopard growls were purchased from the National Sound Archive, London (see 33 for spectrographic illustrations of the playback stimuli). The same visual models (stuffed leopard or crowned eagle) and sound stimuli were used for all tests. All acoustic stimuli were broadcast with a SONY WMD6C Walkman connected to NAGRA DSM speaker-amplifier so that calls sounded natural and could be clearly heard at a distance of about 20 m. The observer then walked with the group and recorded the male's behaviour. Vocal responses were recorded using a SONY TCD–D100 DAT Walkman, a SENNHEISER ME88 microphone, and a LAVALIER microphone for observer comments.

All calls were digitised at a sampling rate of 44.1 kHz, 16 bits accuracy, using Raven 1.3 software, to extract basic measurements, such as the duration and the frequency at which the highest spectral amplitude occurred at the beginning and end of the call unit. We then performed a Pearson's Principal Component Analysis on the entire data set (N = 1,067 calls) to investigate the clustering of the different call stems on the basis of their basic acoustic parameters, i.e. duration, start frequency, and transition frequency. Transition frequency was calculated by subtracting the start from the end frequency of the call stem (fig. 1). To explore the nature of the acoustic differences between the different call types, we conducted analyses of variance, followed by Tukey multiple comparisons post hoc tests, for five major uncorrelated acoustic variables. Caller identity was treated as a random factor. To explore the relation between the different call types and contexts (naturalistic observations and predator simulation experiments), we used generalised linear models (Poisson error structure with log link function, type III; likelihood ratios followed by chi-square tests). For naturalistic observations, the dependent variable was the number of calls produced of each type per male and event category with several occurrences of each event category per male. For the predator experiments, we examined both the effects of predator type and the modality of detection.

In a final analysis, we were interested in how reliable human observers can discriminate the different call types by ear. For this purpose, KO selected a large sample of the original all-occurrence database of the habituated males (N = 877 calls, the remaining 17.8% of the entire dataset were excluded because of substandard recording quality). All sound files were anonymised before classification. The procedure was conducted three times on separate days. Classification of the six call types was highly accurate (reliabilities: 96% between day 1 and 2; 96% between day 1 and 3, and 98% between day 2 and 3), demonstrating that humans can discriminate these six call types very reliably.

## Supporting Information

Audio S1 “Hok” calls are almost exclusively associated with crowned eagle presence.
(0.05 MB WAV)

Audio S2 “Hok-oo” are given to a range of disturbances within the canopy, including eagles, the presence of neighbouring groups and, on a few occasions, to a flying squirrel. While producing these calls, males adopt a threat posture, combined with flashing their eyelids, sometimes combined with a short dash towards the disturbance.
(0.06 MB WAV)

Audio S3 “Krak” calls are exclusively given after detecting a leopard.
(0.09 MB WAV)

Audio S4 “Krak-oo” function as a general alert call and can be given to almost any disturbance.
(0.09 MB WAV)

Audio S5 “Wak-oo” calls are given to the same events as “hok-oo” calls (eagles, other flying animals, Diana monkey eagle alarms), but not to neighbours.
(0.14 MB WAV)

Audio S6 “Boom” calls are given to non-predatory contexts, such as a falling branch or tree, to initiate or halt group travel, during disputes with neighbours, and to any unusual vocal excitation with the group.
(0.20 MB WAV)
